# Apoptotic Cell Death in Neuroblastoma

**DOI:** 10.3390/cells2020432

**Published:** 2013-06-20

**Authors:** Yuanyuan Li, Akira Nakagawara

**Affiliations:** Division of Biochemistry and Innovative Cancer Therapeutics, Chiba Cancer Center Research Institute, 666-2 Nitona, Chuoh-ku, Chiba 260-8717, Japan. E-Mail: liyuan@chiba-cc.jp

**Keywords:** neuroblastoma, spontaneous regression, apoptosis

## Abstract

Neuroblastoma (NB) is one of the most common malignant solid tumors in childhood, which derives from the sympathoadrenal lineage of the neural crest and exhibits extremely heterogeneous biological and clinical behaviors. The infant patients frequently undergo spontaneous regression even with metastatic disease, whereas the patients of more than one year of age who suffer from disseminated disease have a poor outcome despite intensive multimodal treatment. Spontaneous regression in favorable NBs has been proposed to be triggered by nerve growth factor (NGF) deficiency in the tumor with NGF dependency for survival, while aggressive NBs have defective apoptotic machinery which enables the tumor cells to evade apoptosis and confers the resistance to treatment. This paper reviews the molecules and pathways that have been recently identified to be involved in apoptotic cell death in NB and discusses their potential prospects for developing more effective therapeutic strategies against aggressive NB.

## 1. Introduction

Neuroblastoma (NB) is an embryonic malignancy in childhood, which originates from precursor cells of the peripheral (sympathetic) nervous system and usually arises in tissues of the sympathetic nervous system, typically in the adrenal medulla or paraspinal ganglia. The majority of the tumors are sporadic and familial patients occupy only 1–2% of all the cases. The tumor preferentially occurs in young children with a median age at diagnosis of 17 months, and accounts for 15% of all pediatric cancers [1]. 

The most unique characteristic of NB is that the tumors exhibit extremely heterogeneous biological and clinical behaviors. The infant patients frequently undergo spontaneous regression even with metastatic disease to the liver, skin and/or bone marrow. On the other hand, the patients of more than one year of age who suffer from disseminated diseases at diagnosis have a poor outcome despite intensive multimodal treatment [2]. This extreme clinical heterogeneity reflects the complexity of genetic and genomic events related to the development and progression of the disease. Currently, NB patients are stratified into low-, intermediate-, or high-risk categories based on risk assessment of well-defined prognostic factors including the age at diagnosis [3], International Neuroblastoma Staging System (INSS) stage [4], tumor histopathology [5], *MYCN* amplification status [6,7] and tumor DNA ploidy [8]. 5-year survival rates are around 95% for the low-risk group and 80-90% for the intermediate-risk group, respectively, but only 30–50% for the high-risk NBs, according to the information published by the American Cancer Society [9] cite as ref. 

Programmed cell death (PCD) is an essential process that occurs intrinsically throughout life in multicellular organisms. So far, three major modes of PCD have been proposed to exist: apoptosis (type I PCD), autophagy (type II PCD) and necrosis (type III PCD), each of which has distinct morphological features [10,11,12]. Apoptosis is a highly-organized cell suicide mechanism that is initiated spontaneously to eliminate damaged cells in a body without causing damage to surrounding cells. Autophagy is basically a self-degradative process that enables cells to sustain energy metabolism for survival in response to nutrient stress by degrading cytosolic components at the lysosome [13]. Based on recent researches, necrosis has been also regarded as a programmed cell death process in response to various physiological and pathological stimuli such as ischemia and pathogen infection. Unlike apoptosis, necrotic cell death occurs accompanied by loss of membrane integrity and efflux of proteolytic enzymes into extracellular space, as may provoke an inflammatory response in the local cellular environment [11]. Notably, accumulating evidence has documented that there exists crosstalk between these PCD mechanisms, therefore cell death is not a single-mode process but a heterogeneous and complicated process [10,11,12]. 

Apoptotic cell death is mainly executed by two distinct molecular signaling pathways: the death-receptor (extrinsic) pathway and mitochondrial (intrinsic) pathway. Activation of the caspase family of cysteine proteases plays a central role in both pathways. Upon binding of the members of the tumor necrosis factor (TNF) superfamily to their receptors (called the death receptors), initiator caspase 8 is activated which in turn triggers the activation of downstream effector caspases including caspases 3, 6 and 7 as well as the cleavage of Bcl-2 family member Bid to promote cell death. Alternatively, the intrinsic pathway involves caspase 9 which is activated downstream of mitochondrial proapoptotic events including the release of cytochrome c into the cytosol and formation of apoptosome, a cytosolic death signaling protein complex. Activated caspase 9 subsequently initiates the activation of downstream effector caspases to result in cell death (reviewed in [10,11,12]). 

Induction of apoptotic cell death is one of the common mechanisms targeted by current anti-cancer therapies. In clinical practice, selection of NB treatment strategies depends on the risk group factors. For the low- and intermediate-risk NBs, surgery alone or in combination with low or moderate-dose chemotherapy has shown to be effective. Especially for infant patients who are younger than one year of age and have localized tumors, a watch-and-see strategy is appropriate since spontaneous regression usually occurs [14,15]. In contrast, the patients with high-risk diseases are generally treated with intensive multimodal therapy including surgery, high-dose multi-agent chemotherapy and radiotherapy along with autologous stem cell transplantation. Even so, most of these still experience aggressive growth and relapse due to acquirement of resistance to treatment. This indicates that apoptotic signaling is a pivotal mechanism that is effectively activated in infant patients to lead to an excellent prognosis but is defective in high-risk NBs causing a fatal outcome. Therefore, a better and deeper understanding of the molecules and pathways that control apoptotic cell death in NB will expectedly provide new insights into developing more effective therapeutic strategies against aggressive NB. 

## 2. Apoptotic Cell Death in Favorable NB

### 2.1. Neurotrophins, Their Receptors and Spontaneous Regression in Neuorblastoma

Spontaneous regression of NB is a process of PCD which is triggered endogenously to delete tumor cells. This process is very similar to the phenomenon occurring in developing sympathetic neurons, since both developing sympathetic neurons and NB cells which spontaneously regress show nerve growth factor (NGF) dependency for survival. PCD occurring in the spontaneous regression of neuorblastoma has been identified as apoptotic cell death with characteristic features of apoptosis including activation of caspases, induction of apoptosis markers as well as DNA fragmentation [16,17]. In addition, autophagic cell death, another form of PCD, has been observed in the degenerating cells within the tumors identified by mass screening [18], a program which has been carried out for 6-month old infants to detect NB. Moreover, lysosomal cell death mediated by lysosomal-associated protein multispanning transmembrane 5 (LAPTM5) has been recently reported in the regressing area of favorable tumors [19], suggesting that spontaneous regression in NB is a complex process which involves a variety of cell death mechanisms. Considering that there exists a cross-talk between apoptosis and autophagy [20], these cell death mechanisms may act synergistically to eliminate tumor cells and contribute to excellent prognosis. 

Neuorblastoma originates from the sympathoadrenal lineage of the neural crest. During normal nervous system development, apoptosis is an important mechanism for the control of cell number and formation of the highly organized nervous system in the body. Developing sympathetic neurons require the neurotrophic factor NGF for survival and differentiation. Deletion of NGF causes these neurons to undergo apoptotic cell death mediated by the mitochondrial (intrinsic) pathway of caspase activation [21,22,23,24,25,26]. The cloning and identification of the NGF receptors have opened the gate for understanding the signal transduction processes mediated through NGF. The TRK tyrosine kinase receptor A (TrkA) as well as p75^NTR^ have been both identified as high and low affinity receptors for NGF, respectively [27,28,29,30]. The NGF dimer binds to a dimeric form of TrkA or a monomeric p75^NTR^ to transduce functional signals and induce neuronal differentiation [31]. 

The Trk tyrosine kinase receptor family is composed of three members: TrkA, TrkB, and TrkC, which are activated by binding to their preferred neurotrophins to manipulate the survival and differentiation of neurons during development. The main ligands for these receptors have been identified as NGF for TrKA, brain-derived neurotrophic factor (BDNF) and neurotrophin-4/5 (NT-4/5) for TrkB, and neurotrophin-3 (NT-3) for TrkC [2]. TrkA and TrkC are both expressed at high levels by many peripheral nervous system neurons at the time of target innervations, while TrkB and its neurotrophin ligand BDNF are mainly expressed in the central nervous system. A recent study using mouse embryonic stem (ES) cells engineered to express the three Trk receptors has shown that TrkA and TrkC but not TrkB instruct developing neurons to die, and the death of neurons is prevented by the addition of their ligands, NGF or NT-3, respectively [32], indicating an important role for NGF/TrKA and NT-3/TrkC signaling in cell number control and the modeling of developing sympathetic and sensory neurons. 

Similar NGF-dependent phenomena are observed in the NBs with favorable prognoses. Both TrkA and p75^NTR^ are expressed at significantly high levels in favorable NBs that express a very small amount of NGF [33,34]. The favorable NB cells expressing TrkA and p75^NTR^ in primary culture are dependent on NGF for survival and differentiation. Once NGF becomes deficient, however, apoptotic cell death is triggered [33]. In contrast to the expression pattern of NGF/TrkA in favorable NBs, TrkB as well as its ligands BDNF and NT-4/5 are preferentially expressed in aggressive NBs in an autocrine and/or paracrine manner [35]. Moreover, expression of TrkB strongly correlates with *MYCN* amplification, a hallmark of aggressive NB [35]. TrkC however is expressed along with TrkA in favorable NBs that express very limited amount of NT-3 [36,37]. Notably, TrkC has been recently identified to behave as a dependence receptor, which induces caspase 9-dependent apoptotic death in the absence of its ligand NT-3 through caspase cleavage-mediated release of an intracellular fragment [38]. Moreover, NT-3 is upregulated in a large fraction of aggressive human neuroblastomas and therefore blocks TrkC-induced apoptosis in human NB cell lines [39]. In addition, the NGF receptor p75^NTR^ is already regarded as a dependence receptor for transducing apoptotic signal when in lack of NGF [40]. Although the molecular mechanism of spontaneous regression in NB is still enigmatic, these studies indicate that the quantitative relationships between NGF/NT-3 and their receptors within the tumorous tissue as well as the acquisition of NGF/NT-3 dependency may be crucial for inducing regression of NB. In response to a microenvironment with limited amounts of NGF/NT-3, NB tumor cells undergo spontaneous apoptosis, thus disrupting the signals transduced via NGF/NT-3 and their receptors may be a promising therapeutic strategy for the treatment of NB. 

### 2.2. p53 Family and Relative Genes

The p53 family consists of three members p53, p73 and p63, which share similar structures including a transactivation domain, a DNA-binding domain and an oligomerization domain, and act as transcription factors. These family members execute critical roles in many important cellular processes such as DNA damage repair, cell cycle regulation, cellular differentiation and apoptosis [41,42]. Due to multiple splicing and alternative promoters, the p53 family members express multiple isoforms containing different domain structures [43]. Of these, amino-terminal truncated forms without the typical transactivation domain (ΔN isoform) exist for each member and commonly act as dominant-negative inhibitors of the full-length proteins. Since the mutation in the p53 gene is the most frequent genetic event in various human cancers, p53 is regarded as a classical tumor suppressor whose inactivation is associated with tumorigenesis. 

Accumulating evidence indicates the involvement of the p53 family in the neuronal death of developing sympathetic neurons. p53 has been shown to play a proapoptotic role in developing sympathetic neuron death regulated via the TrkA and p75 neurotrophin receptors. In cultured neonatal sympathetic neurons, p53 protein levels are elevated in response to both NGF withdrawal and p75^NTR^ activation. NGF withdrawal also results in elevation of Bax, a direct apoptotic target of p53 [44]. Moreover, overexpression of p53 is sufficient to induce apoptosis of postmitotic sympathetic neurons in the presence of NGF [45], while embryonic sympathetic superior cervical ganglia (SCG) neurons isolated from p53^−/−^ mice exhibit enhanced survival in culture even in the absence of NGF [46]. These *in vivo* and *in vitro* findings document that p53 is essential in an apoptotic signaling cascade that is activated following NGF depletion. In addition, p63 has been reported to play an essential proapoptotic role during naturally occurring neuronal death [47]. Sympathetic neurons express the full-length TAp63γ isoform during the developmental death period, and its levels increase following NGF withdrawal. Like p53, overexpression of TAp63γ causes neuronal apoptosis in the presence of NGF accompanied with induction of Bax, the common apoptotic target of the p53 family, while cultured p63^−/−^ neurons are resistant to apoptosis induced by NGF withdrawal. Interestingly, TAp63 triggers neuronal death in the absence of p53, whereas p53 requires coincident p63 expression for its proapoptotic functions, implying a dominant and independent role of p63 in developmental sympathetic neuron death. On the other hand, in contrast to p53 and p63, p73 functions as an anti-apoptotic protein in this process, although it has a similar ability with its siblings p53 and p63 to induce apoptosis in non-neuronal cells [48,49]. ΔNp73, a truncated form of p73 lacking the transactivation domain, is predominantly expressed in developing neurons whose levels are dramatically decreased when sympathetic neurons undergo apoptosis in response to NGF withdrawal, while its increased expression rescues these neurons from apoptosis induced by NGF deprivation or p53 overexpression [50]. In p73^−/^^−^ mice, the apoptosis of developing sympathetic neurons is greatly enhanced. Correspondingly, ΔNp73 promotes the survival of sympathetic neurons during the developmental period of naturally occurring neuronal death via p53-dependent and/or p53-independent mechanisms [51]. Thus, truncated p73 serves as an apoptosis antagonist of p53 (and perhaps of p63) during the development of sympathetic neurons. Notably, p63 as well as p73 has been recently implicated to serve in the maintenance of neural stem cells [52,53,54], whereas p53 acts in the opposite way in the self-renewal of adult neural stem cells [55]. 

To date, no direct evidence has yet been proposed to implicate p53 family members as essential players in the spontaneous regression of NB, as they do in developing sympathetic neuron death. However, considering the following facts (i) the mutation of p53 is rare in primary NBs; (ii) p53 is induced and p53-dependent apoptotic pathway is activated in response to chemotherapeutic agents in NB murine models [56] as well as NB cell lines, and (iii) exogenous expression of TrkA induces p53-mediated apoptosis even in the NB cells with *MYCN* amplification, which is inhibited by p53 inactivation [57], p53 should be of functional importance and contribute to apoptotic cell death in favorable NB. Besides, several lines of evidence have documented that p73 is involved in induction of apoptosis and differentiation in NB cells, two important processes that lead to a favorable outcome. Overexpression of p73 induces apoptosis in several NB cell lines [58], and inhibits *MYCN* expression in NB cells at both transcriptional and protein levels [59]. Moreover, p73 itself has an ability to induce neuronal differentiation in NB cells [60]. Expression levels of p73 increase in NB cells treated by retinoic acid (RA), an agent with the potential to induce neuronal differentiation whose derivative13-*cis*-retinoic acid has been clinically administrated in NB treatment [61]. It is notable that the p73 gene locus maps to 1p36.3, a region frequently deleted in high-risk NBs. However, expression levels of ΔNp73 are significantly higher in aggressive NBs and independently predict poor prognosis [62,63]. ΔNp73 has been reported to have anti-apoptotic activity and inhibit neuronal differentiation in NB cells, at least in part by competitively restraining the transactivation activity of full-length p53 and p73 [64,65]. These findings denote that imbalance between p53 and p73 *versus* antagonistic ΔNp73 inactivates p53/p73-mediated proapoptotic and pro-differentiational signaling pathways in high-risk NBs. On the other hand, p73 is a direct transcription target of E2F1 and essential for the E2F1-induced p53-independent apoptotic pathway [66]. E2F1 has been shown to be involved in the regulation or induction of NGF-depletion-induced PCD of sympathetic neurons [67,68]. Given the fact that the patients with relapsed NBs gradually acquire p53 mutations and become resistant to chemotherapies while p73 is rarely mutated in human cancer, the strategic targeting of the p73-mediated apoptotic pathway might be of therapeutic benefit. A recent study using an antisense oligonucleotide against ΔNp73 mRNA was reported to successfully induce apoptosis in NB cells through the upregulation and activation of the p73 downstream targets PUMA and Bax [69]. 

Microarray-based gene expression profiling has been widely used to identify the prognosis-related genes in various human cancers including NB. To determine the different gene signatures expressed between favorable and unfavorable subsets, we have developed a cDNA chip harboring 5340 NB-derived genes. Gene expression analysis of 136 NBs has enabled us to identify a group of genes which are preferentially expressed in favorable or unfavorable NBs and predict the prognosis with high efficiency and reproducibility [70]. Of those highly expressed in the favorable subset, several genes have been demonstrated to be involved in the p53-mediated apoptotic cell death pathway by gene function approaches. NEDD4-like ubiquitin protein ligase-1(NEDL1/HECW1) is one of such genes which interacts with p53 and enhances the p53-mediated apoptosis in NB cells [71]. Of note, NEDL1 can bind and ubiquitinate mutant superoxide dismutase-1 (SOD1), the causal protein of familial amyotrophic lateral sclerosis (FALS), as results in the formation of potentially cytotoxic protein that aggregates in the spinal cord and consequent motor neuron death in FALS [72]. Correspondingly, several lines of evidence have indicated the involvement of p53 in neuronal degenerative conditions including ALS [73]. On the other hand, NEDL1 also targets Dishevelled-1, one of the key transducers in the Wnt signaling pathway, for ubiquitin-mediated degradation [72], implicating NEDL1 as a possible negative modulator of the Wnt signaling. 

Additionally, UNC5D, a member of the netrin-1 dependence receptor UNC5 (also called UNC5H) family, has been selected from our NB cDNA project as a highly expressed gene in favorable NBs. Dependence receptors transduce two opposite intracellular signals depending on the availability of their ligands. In the presence of ligand, these receptors transduce a positive signal for survival, differentiation or migration. Conversely, in the absence of ligand, the receptors conduct a negative signal for apoptosis [74,75]. A putative caspase 3/7 cleavage site exists in the intracellular domains of most of the dependence receptors identified so far. The possible mechanism of the dependence receptors-mediated apoptotic signaling is considered to be related to the cleavage of their intracellular fragments by caspase. Likewise, UNC5D possesses a caspase 3/7 cleavage site in its intracellular domain and is highly expressed in favorable NBs that express netrin1 at very low levels, as facilitates UNC5D to transduce proapoptotic signals through cleavage and release of its intracellular fragment and therefore is associated with favorable prognosis [76]. On the other hand, the other family members of UNC5 (UNC5A, UNC5B and UNC5C) are also expressed in NB, however their expression levels show no correlations with favorable outcome [76]. Intriguingly, UNC5B and UNC5D have been identified to be direct transcriptional targets of p53 and to contribute to the p53-dependent apoptotic response [77,78]. Deleted in colorectal carcinoma (DCC) is another netrin1 dependence receptor that has been regarded as a tumor suppressor and is inactivated in various cancers. Loss of DCC expression is associated with disease dissemination in NB independently of *MYCN* amplification [79,80]. On the other hand, netrin1, the ligand for the UNC5 family and DCC, is robustly expressed in aggressive stage 4 NB cells in an autocrine manner and confers a selective advantage for tumor growth and metastasis [81]. Thus, netrin1 and its dependence receptors UNC5 and DCC seem important for cell fate determination in NB. Given that disruption of the autocrine loop of netrin1 by an antagonistic extracellular fragment of netrin1 which blocks the function of netrin1 triggers NB cell death and inhibits NB metastasis in avian and mouse models [81], interference with the netrin1 signaling could provide an alternative promising therapeutic strategy against aggressive NBs. 

### 2.3. Candidate Tumor Suppressor Genes in 1p36 Related to Neuroblastoma

Deletion of a distal part of chromosome 1p (1p36) is one of the most common chromosomal aberrances observed in NB tumors and associated with a poor prognosis, suggesting that this region should harbor one or more tumor suppressor genes closely related to NB development. Besides p73, several important tumor suppressor genes such as *KIF1Bβ*, *RUNX3* and *CHD5* have been identified in this region so far. 

#### 2.3.1. KIF1Bβ

We and others have previously identified a 500-kb homozygous deletion at 1p36.2 harboring at least six genes, *PEX14*, *UFD2a*, *KIF1B*, *CORT*, *DFF45*, and *PGD*, in a NB-derived cell line NB1/C201 [82,83,84]. Of those, KIF1Bβ is expressed at high levels in favorable NBs but is markedly down-regulated in advanced stages of NB tumors, and its hemizygous deletion is significantly correlated with advanced stages and *MYCN* amplification [85]. Enforced expression of KIF1Bβ results in an induction of p53-independent apoptotic cell death in association with G_2_-M phase arrest, whereas its knockdown led to an accelerated cell proliferation as well as enhanced tumor formation in nude mice [85]. KIF1Bβ is a member of the kinesin superfamily proteins which are involved in the transport of organelles, vesicles, protein complexes, and RNA to specific destinations [86,87]. Intriguingly, KIF1Bβ acts downstream from EglN3 and is both necessary and sufficient for neuronal apoptosis when NGF becomes limiting [88]. EglN3 is a prolyl hydroxylase that acts as a downstream effector of c-Jun to mediate PCD during developmental apoptosis of normal sympathetic neuronal precursors triggered by NGF withdrawal [89]. The products of *VHL*, *NF-1*, *c-Ret*, and Succinate Dehydrogenase Subunits B and D (*SDHB* and *SDHD*) genes regulate this process. Notably, germline mutations in these genes cause familial pheochromocytoma [90,91], an adrenal medullary tumor that is derived from sympathetic neuronal precursor cells of the neural crest. The mutants block the apoptotic cell death induced by c-Jun/EglN3 through upregulating c-Jun antagonist JunB as well as inactivation of the succinate dehydrogenase activity of SDH, which is required by EglN3-induced neuronal apoptosis [89]. On the other hand, inherited loss-of-function *KIF1Bβ* missense mutations have been identified in NBs and pheochromocytomas and an acquired loss-of-function mutation in a medulloblastoma [88]. Altogether these studies support the view that the proapoptotic KIF1Bβ is necessary for the natural neuronal death occurring in developing sympathetic neurons after NFG withdrawal, and its inactivation due to deletion and/or mutation facilitates the generation of sympathetic neuronal precursor cells-derived tumors including NB and pheochromocytoma. As for the other genes identified in the 1p36.2 region, no evidence has yet been presented to confirm that they are involved in apoptotic cell death in NB, although expression levels of PEX14 and UFD2a are higher in favorable NBs as compared to unfavorable ones [83,92] and a splice site mutation is detected in the *UFD2* gene in a stage 3 NB with a fatal outcome [92]. 

#### 2.3.2. RUNX3

The Runt-related transcription factor 3 (RUNX3) gene is localized at chromosome 1p36. This gene encodes a product which acts as a transcriptional factor with tumor suppressive function. So far, inactivation of RUNX3 caused by hemizygous deletion, promoter hypermethylation, or protein mislocalization is frequently observed in various human solid tumors, especially in gastric cancers [93]. Although the detailed molecular mechanisms underlying the RUNX3 tumor suppressive function are still unclear, several lines of studies have provided the evidence that RUNX3 has an ability to regulate both transforming growth factor beta (TGFβ) and Wnt signaling pathways. On one hand, RUNX3 can cooperate with the TGFβ signaling effector Smad to induce the expressions of p21^WAF1^ as well as proapoptotic gene Bim1 in response to TGFβ [94,95]. On the other hand, RUNX3 attenuates the Wnt signaling through forming a ternary complex with β-catenin/TCF4, which inhibits the DNA binding and transcriptional activities of β-catenin/TCF4 [96]. Recently, our group has shown that RUNX3 promotes the phosphorylation of p53 at Ser15 and therefore enhances the p53-dependent apoptosis during a DNA damage response [97]. 

On the other hand, RUNX3 plays a pivotal role in the development of proprioceptive sensory neurons. RUNX3 represses transcription of the neurotrophine receptor TrkB in dorsal root ganglion (DRG) neurons during neuronal fate determination [98], and controls the axonal projection of these neurons to their central and peripheral targets [99]. In NB, deletion of the *RUNX3* locus is commonly observed in aggressive NBs, especially in those with *MYCN* amplification, implicating that loss of RUNX3 expression might be related to tumorigenesis and progression in NB. 

#### 2.3.3. CHD5

The chromodomain helicase DNA-binding protein 5 (CHD5) gene resides in 1p36.31. CHD5 belongs to the chromodomain superfamily of proteins and has been proposed to function as a chromatin remodeling protein. A study using the chromosome engineering approach to generate mouse models with gain and loss of a region corresponding to human 1p36 has documented that CHD5 functions as a tumor suppressor *in vivo* that controls proliferation, apoptosis, and senescence via regulating the p19(Arf)/p53 pathway [100]. Consistently, heterozygous deletion of the *CHD5* gene as well as the promoter CpG island hypermethylation is frequently observed in various human cancers [101,102], suggesting that inactivation of CHD5 caused by deletion and epigenetic silencing commits to tumorigenesis and tumor progression. 

A quantitative investigation of mRNA level attests that CHD5 is preferentially expressed in the nervous system including total brain, fetal brain, and cerebellum but is undetectable in almost all other tissues, except for a moderate expression in the adrenal gland [103]. Recently, expression of the CHD5 protein has been further confirmed by immunohistochemical analysis to be limited to neuronal cells of brain cortex, cerebellum, spinal cord and sympathetic ganglion [104]. This neuron-specific expression pattern suggests that CHD5 may play an important role in normal neuronal development and function. 

In NB, several independent studies have reported that high CHD5 expression is strongly correlated with favorable clinical/biological features and outcome [104,105]. Like most of the other 1p36 tumor suppressor genes identified, somatically acquired *CHD5* mutations are rare in primary NBs, however methylation of the *CHD5* promoter is a common event in the high-risk tumors as well as NB cell lines, and it is generally associated with both 1p deletion and *MYCN* amplification [105,106]. According to the results from Lavarino’s group, the most intense expression of CHD5 is observed in stage 4s NBs amongst all the stages. Moreover, CHD5 is restricted to differentiating neuroblasts and ganglion-like cells, and is absent in undifferentiated neuroblasts and stromal Schwann cells within NB tissues [104]. The stage 4s patients are generally infants who have a high incidence of spontaneous regression even with widespread diseases [107]. Their findings strongly suggest that CHD5 may play a role in the induction of apoptotic death and differentiation in NB, which contribute to a favorable outcome. Elucidation of the molecular mechanism by which CHD5 modulates these processes will expectedly open new avenues for targeted cancer therapy in NB. 

### 2.4. Other Genes

Screening of primary NBs using our NB-specific cDNA chip has identified the novel gene BCH motif-containing molecule at the carboxyl terminal region 1 (BMCC1) as one of the genes highly expressed in favorable NBs and associated with favorable prognosis. In primary culture of newborn mice superior cervical ganglion (SCG) neurons, BMCC1 expression is downregulated after NGF-induced differentiation, but upregulated during the NGF depletion-induced apoptosis [108]. Also, induction of endogenous BMCC1 is observed in the NB cells undergoing apoptosis after treatment with retinoic acid, and the SCG neurons obtained from newborn *BMCC1* transgenic mice become more sensitive to depletion of NGF than that from wild type mice. These findings argue that BMCC1 might play a role in NGF deficiency-induced neuronal death and therefore contribute at least in part to both developing sympathetic PCD and also spontaneous regression of NB.

We and others have found that the expression of the tumor suppressor in lung cancer 1 (TSLC1, also designated as immunoglobulin superfamily 4 (IGSF4), and cell adhesion molecule 1 (CADM1)) is remarkably downregulated in unfavorable NBs [109,110]. To date, the silencing of *TSLC1* has been observed in a number of human cancer tissues including lung, prostate, liver, stomach, pancreatic, and breast cancers, mainly due to hypermethylation of its promoter region. In NB, we have found that 35% of primary NBs harbor loss of heterozygosity (LOH) on *TSLC1* locus [109], although hypermethylation in the *TSLC1* promoter region is undetectable [109,110]. Several lines of evidence have indicated that TSLC1 has antiproliferative and proapoptotic activities [111,112], as is also verified in NB cells [109,110]. It will be of interest to investigate the molecular mechanism underlying TSLC1-mediated growth arrest and cell death. 

Our NB cDNA project has enabled us to identify two members of the human neuronal leucine-rich repeat protein (NLRR) family, NLRR1 and NLRR3, with different expressions between favorable and unfavorable subgroups [113]. In contrast to NLRR1 that is highly expressed in unfavorable NBs with *MYCN* amplification [114], NLRR3 is predominantly detected in the favorable subgroup [113,115]. Correspondingly, expression of the mouse counterpart of *hNLRR3* is up-regulated after NGF-induced differentiation but down-regulated after NGF depletion-induced apoptosis in newborn mouse SCG neurons [113]. Similar results are observed in human NB cells induced to differentiate by retinoic acid, accompanied with reduced expression of MYCN, suggesting that NLRR3 may be essential in differentiation [115]. Indeed, NLRR3 is a direct target of MYCN, which is negatively regulated by the MYCN/Miz-1 transcriptional complex [115]. On the other hand, NLRR1 is also a direct target of MYCN that nevertheless enhances EGF-mediated MYCN induction and accelerates tumor growth *in vivo* [116], exhibiting a biological function opposite to its homologue NLRR3 in NB. Given that the NLRR family is a type-1 transmembrane protein, NLRR1 may be potentially an attractive target for therapeutic intervention in NB. 

In addition, several genes have been reported so far by other groups to be associated with favorable prognosis in NB. ZNF423 has been identified as a prognostic biomarker for NB independent of *MYCN* amplification, whose low expression predicts poor outcome [117]. Remarkably, ZNF423 acts as a critical transcriptional coactivator of the retinoic acid receptors and is required for RA-induced neuronal differentiation [117]. More recently, Bernards’s group has identified the functional role of tumor suppressor NF1 in retinoic acid-induced differentiation in NB. Loss of NF1 activates RAS-MEK signaling, which in turn represses ZNF423 [118]. Consistently, the NB patients with low expressions of both NF1 and ZNF423 have an extremely poor outcome. Of interest, inhibition of the MEK kinase with a small-molecule inhibitor restores both ZNF423 expression and sensitivity to RA in NF1 knocked down cells, suggesting the therapeutic possibility for overcoming the RA resistance encountered in NB clinical practice. On the other hand, the non-receptor tyrosine kinase Fyn has been found to be associated with favorable NBs. High expression of Fyn is prognostic of long-term survival of NB patients independently of *MYCN* amplification. Active Fyn kinase induces cell cycle arrest and differentiation in NB cells [119], therefore downregulation of Fyn in advanced NBs may promote the development of tumors. These data prove that the anti-tumor effect of these genes is mainly attributed to their abilities to induce neuronal differentiation in NB. No evidence has yet been presented to show that they are involved in induction of apoptosis. 

## 3. Aberration of Apoptosis-Related Molecules/Pathways in Unfavorable NB

### 3.1. Caspase 8 and Death Receptor Signaling Pathway

Caspase 8 is a key regulator in apoptosis induction through the death receptor (extrinsic) pathway. In response to extrinsic stimuli, caspase 8 is activated by dimerization and subsequent cleavage to initiate the apoptosis signaling cascade. However, loss of caspase 8 expression is commonly observed in primary NBs. So far several lines of evidence have indicated that the absence of caspase 8 expression may be attributed to genomic and/or epigenetic aberrance. Homo- or heterozygous genomic deletion in the *CASP8* gene has been found in some NBs [120]. More frequently, hypermethylation of the regulatory region in the *CASP8* gene has been identified to account for decreased caspase 8 expression [120,121,122], and to be associated with poor outcome in NB patients [120,122,123]. Furthermore, some studies indicate that loss of caspase 8 expression is correlated with *MYCN* amplification [120,124]. However, a recent study of 162 patients shows that loss of caspase 8 expression is correlated with neither *MYCN* amplification nor poor prognosis in NB [125]. In addition to genomic and epigenetic mechanisms, inactivation of caspase 8 in NB may be also caused by alternative splicing of intron 8 in the *CASP8* gene, which produces caspase 8L, an isoform lacking caspase activity and therefore acting as a dominant negative inhibitor of wild-type caspase 8 [126]. 

Besides caspase 8, Fas receptor CD95 as well as tumor necrosis factor-related apoptosis-inducing ligand (TRAIL) receptors DCR1 and DCR2 have been found to be hypermethylated in their promoter regions in NB [127,128], leading to tumor-specific downregulation of these genes and therefore impairing apoptosis signaling via death receptor pathway in NB. On the other hand, the cytokine INFγ has been found to enhance the susceptibility to apoptosis in NB through upregulation of caspase 8 [129,130]. Nevertheless, induction of caspase 8 by INFγ is not sufficient for most TRAIL-resistant NB cells to undergo apoptosis, while chemotherapy in combination with INFγ and TRAIL treatment dramatically restores apoptosis sensitivity even in chemo- and TRAIL- resistant NB cells by synergistic induction of both caspase 8 and TRAIL receptors [129,130]. Together these imply the complexity of death receptor pathway defects as well as the potential value of the combined therapeutics in aggressive NBs.

### 3.2. MYCN and its Role in Apoptosis Regulation

MYCN is predominantly expressed in the central and peripheral nervous systems during the early stages of embryonic development and plays a pivotal role in regulation of cell growth, differentiation and apoptosis. Given the facts (i) MYCN amplification is a hallmark of high-risk NBs strongly predicting poor outcome and (ii) *MYCN*-trangenic mice spontaneously develop NB [131], MYCN has been regarded as a driver of NB tumorigenesis and progression. 

MYCN acts as a transcriptional factor to execute its biological functions by directly or indirectly regulating the transcriptions of its downstream target genes. In NB, MYCN is thought to play a dual role in cell fate control. On one hand, increased MYCN expression promotes cell proliferation by up or down regulating the cell cycle related genes including *SKP2*, *NLRR1*, *E2F1*, *ODC*, *Id-2*, *p21^WAF1^* and *DKK3* [132]. Recently, a study conducted by one of our groups identified the polycomb protein Bmi1 as a novel direct transcription target of MYCN. Upregulation of Bmi1 by MYCN accelerates cell growth and inhibits cell differentiation through the Bmi1-dependent repression of KIF1Bβ and TSLC1 [133]. In addition, MYCN directly transactivates the *MRP1* gene, which encodes a multidrug resistant protein and may confer the resistance of NB cells to chemotherapy [134]. 

On the other hand, MYCN expression increases the cell sensitivity to apoptosis. In response to DNA damage, both *MYCN*-amplified NB cells and those with single copy *MYCN* but ectopically expressed MYCN are more prone to undergo apoptosis [135,136]. Remarkably, tumor suppressor p53 is a direct target of MYCN [137]. MYCN sensitizes NB cells to apoptosis by transcriptional activation of p53 as well as its proapoptotic targets PUMA and Bax [137,138]. Nevertheless, MYCN attenuates the p53 function by upregulating MDM2, a p53 negative regulator [139], as well as TWIST, an oncogenic transcriptional factor that suppresses the p14^ARF^-p53 mediated apoptosis pathway [140]. In addition, several lines of evidence have shown that MYCN sensitizes NB cells to apoptosis by activation of the extrinsic apoptosis pathway [141,142]. This process requires the activation of caspase 8. Considering that loss of caspase 8 expression is commonly observed in NBs, and anti-apoptotic proteins such as Survivin and Bcl-2 are abnormally high in aggressive NBs, the resistance of NBs with *MYCN* amplification may be attributed to dysfunction of apoptotic pathways.

### 3.3. Wnt Signaling Pathway

Wnt signaling is a key pathway in embryonic development and regulates a wide range of biological processes including cell fate, proliferation, specificity and polarity. Aberrant Wnt signaling underlies various human diseases, in particular cancer [143,144]. As for NB, the Wnt/β-catenin canonical pathway has been shown to be deregulated and to account for the malignant phenotype and chemoresistance in a subset. According to the study by Hogarty’s group, aberrant activation of β-catenin canonical signaling is observed in both primary NBs and NB cell lines without *MYCN* amplification, which therefore leads to the high expression of its downstream target c-Myc, another member of the MYC oncogenic family [145]. Additionally, a recent study using gene expression profiling has indicated that the upregulation of the Wnt receptor FDZ1 in Doxorubicin-resistant NB cells mediates sustained activation of the Wnt/β-catenin pathway and confers chemoresistance by upregulating the *MDR1* gene that encodes a multidrug resistance protein [146]. More recently, Lyman’s group has reported that β-catenin canonical signaling is aberrantly activated in the NB cells expressing CD133, a candidate marker for cancer stem cells, as is correlated with a resistant phenotype in those cells [147]. Interestingly, addition of the Wnt inhibitors restores the sensitivity of CD133-positive NB cells to Doxorubicin, suggesting the potential of targeting the Wnt/β-catenin pathway for effective therapy in aggressive NBs. 

On the other hand, low expression of Wnt-5a has been observed in high-risk NBs with poor outcome [148]. Wnt-5a is a member of the Wnt ligand family, which transduces the non-canonical Wnt/calcium signal involved in patterning decisions in the embryonic nervous system [149]. Surprisingly, treatment with retinoic acid induces differentiation in metastatic NBs, accompanied with the upregulation of Wnt-5a and PKC-θ, but without activation of the Wnt/β-catenin pathway [148,150], implicating the involvement of the Wnt/calcium signaling pathway in response to RA in disseminated disease. 

### 3.4. ALK

Anaplastic lymphoma kinase (ALK) is an orphan receptor tyrosine kinase that belongs to the insulin receptor superfamily. So far, genomic and genetic aberrances of the *ALK* gene have been found to be correlated with tumorigenesis and progression in a variety of human malignancies. These aberrances include structural rearrangement (translocation) for the generation of oncogenic ALK fusion protein, *ALK* gene amplification and mutation [151]. In NB, germ-line mutations in the *ALK* gene have been described in familial NB patients and thus *ALK* is regarded as a major familial NB predisposition gene [152]. Also, we and others have identified somatic *ALK* mutations or amplifications in 6–11% of the sporadic cases, who commonly have metastatic diseases and *MYCN* amplification [153,154,155,156]. Most reported *ALK* mutations are localized in the tyrosine kinase domain and are speculated to generate constitutively activated proteins [157]. In the context of tissue types and *ALK* gene aberrance patterns, aberrant activation of ALK has been assumed to initiate diverse downstream signaling pathways that control cell proliferation, survival and cell cycle, which consequently leads to cellular transformation [151]. Encouragingly, inhibition of ALK by small molecule tyrosine kinase inhibitors induces apoptosis and cell cycle arrest [151,157]. Although the NB cells harboring different types of *ALK* mutations are not identically sensitive to the inhibitors available at present [158], the results inspire the development of novel antitumor compounds and more effective therapeutic strategy targeting ALK for the patients with mutated *ALK*. Indeed, Phase I/II clinical trials of the ALK inhibitor crizotinib for NB are ongoing in the USA. 

### 3.5. The Bcl-2 Family

The Bcl-2 family proteins are key regulators of apoptosis through the mitochondrial (intrinsic) apoptotic pathway. The Bcl-2 family is composed of anti-apoptotic members (Bcl-2, Bcl- _X_L, Mcl-1, *etc*.) as well as pro-apoptotic members including the BH multi-domain subgroup (Bax, Bak and Box) and the BH3-only proteins (Bim, Bid, Bad, Bik, Puma, Noxa and Hrk). The balance between anti-apoptotic and pro-apoptotic members has been proposed to be crucial for controlling the activation of the intrinsic apoptotic pathway [159,160]. 

In NB, enhanced expression of anti-apoptotic Bcl-2 is observed in both primary NB tissues and NB cell lines [161,162,163]. Also, a recent study showed that anti-apoptotic Bcl-2 family member Mcl-1 is expressed at high levels in NB tumors with unfavorable biology, while pro-apoptotic members of the mitochondrial apoptotic pathway are expressed to a lower extent in these tumors [164]. Additionally, another anti-apoptotic member Bcl- _X_L is highly expressed in most NB cell lines [165]. Moreover, exogenous expression of Bcl-2 or Bcl- _X_L renders the NB cell lines resistant to chemotherapy-induced apoptosis [165,166]. These results indicate that a relatively high ratio of anti-apoptotic members *versus* pro-apoptotic members of the Bcl-2 family suppresses the mitochondrial pathway-mediated apoptosis and favors survival of tumor cells in NB. More recently, several studies provided evidence that diverse expression profiles of the Bcl-2 family account for the heterogeneity of chemo-resistance in NB [167,168], implicating that individualized selection of small molecule inhibitors targeting to special anti-apoptotic Bcl-2 members may be important for treating chemo-resistant NB patients. Notably, 13-*cis*-retinoic acid, a drug commonly used for high-risk NB, can reduce apoptosis mediated by several chemotherapeutics through upregulating Bcl-2 and Bcl-_X_L, when applied in combination with those agents in several NB cell lines [169]. 

### 3.6. Survivin

As the smallest member of the Inhibitor of Apoptosis (IAP) gene family, survivin has been denoted to play a pivotal role in many cellular processes encompassing cell survival, cell division, cell stress response and apoptosis resistance. In most normal adult tissues, the *survivin* promoter activity is basically silent so that its expression is undetectable or at very low levels. Conversely, survivin is predominantly overexpressed in various human cancers and is consistently associated with disease progression, metastasis, resistance to therapy and poor outcome. To date, accumulating evidence has revealed that many oncogenic transcriptional factors including STAT3 [170], c-Myc [171], Notch [172], β-catenin/TCF-4 [173], and NFκB [174] directly or indirectly transactivate the *survivin* promoter. On the other hand, several tumor suppressors such as p53 [175,176], Rb [177], BRCA1 [178] and APC [179] repress its expression at the transcriptional level. Thus, it has been proposed that tumor-specific increased expression of survivin may be due to the aberrant activation of those oncogenic pathways and/or the inactivation of those tumor suppressors [180]. 

The *survivin* locus is mapped to 17q25, a region frequently amplified in NB. Accordingly, we and others have found that survivin is expressed at high levels in aggressive NBs and is remarkably associated with high-risk factors including advanced stage, older age and low levels of TrkA expression, as well as unfavorable prognosis [181,182,183,184]. Intriguingly, it has been reported that a survivin minigene DNA vaccine is effective to suppress tumor growth and metastasis in a syngeneic NB mouse model [185], providing a promising approach to the development of a novel therapeutic strategy against NB. 

### 3.7. The LMO Family

The LIM-only proteins (LMO) family consists of LMO1, LMO2, LMO3, and LMO4. These members possess two highly conserved cysteine-rich zinc finger–like LIM domains and are involved in cell fate determination and differentiation during embryonic development. Several lines of evidence suggest that LMO1 and LMO2 play oncogenic roles in T-cell acute lymphoblastic leukemia [186,187]. Recently, LMO4 has been implicated as a contributor to the genesis and development of breast cancer [188,189]. 

Previously, we have found that LMO3 is expressed at significantly high levels in human unfavorable NBs and is associated with a poor prognosis [190]. Moreover, LMO3 exhibits an oncogenic potential in view that its overexpression promotes proliferation of NB cells *in vivo* and *in vitro*. Although LMO proteins lack DNA-binding activity, accumulating evidence suggests that LMO proteins are involved in transcriptional regulation of specific target genes in collaboration with other transcription factors. With regard to NB, our study has elucidated that LMO3 forms a nuclear complex with neuronal-specific basic helix-loop-helix (bHLH) transcription factor HEN2, which inhibits another bHLH protein HES1 and consequently leads to upregulation of Mash1, a proneural bHLH transcription factor that is negatively regulated by HES1 and plays a critical role in the development of sympathetic neurons [191]. Consistently, expression levels of HEN2 and Mash1 are significantly higher in unfavorable NBs [190,192,193]. On the other hand, we have also demonstrated that LMO3 is a direct interacting partner of p53 [194]. Interaction of LMO3 with p53 suppresses transactivation activity of p53. Therefore, overexpressed LMO3 seems to contribute to an aggressive phenotype through different mechanisms in NB. 

Recently, a genome-wide association study (GWAS) has identified *LMO1* as an oncogene of NB [195]. According to their results, the aberrance in the *LMO1* locus is found in 12.4% of NB patients and is associated with more advanced diseases. Moreover, the germline single nucleotide polymorphism (SNP) risk alleles and somatic copy number gains are associated with increased LMO1 expressions in both NB cell lines and primary tumors, consistent with a gain-of-function role in tumorigenesis. 

## 4. Conclusions

Defects in apoptotic regulatory machinery confer tumor cell growth advantages and therapeutic resistance. The accumulated evidence described above manifests the existence of heterogeneous and collective aberrations in apoptotic regulation pathways in NB that lead to resistant behaviors and poor outcomes. In spite of the remarkably improved clinical response rate to current therapeutics in NB, the majority of patients with advanced stages still undergo relapse and have a 5-year event-free survival of <50%. The complexity of the abnormality occurring in aggressive NBs implicates that it will be important and imperative to identify new targets in the context of personal conditions for drug discovery. Development of personalized therapy will be expected to provide more effective therapeutic strategies against high-risk NBs for long-term survival. 
